# Decoupled Classifier Knowledge Distillation

**DOI:** 10.1371/journal.pone.0314267

**Published:** 2025-02-21

**Authors:** Hairui Wang, Mengjie Dong, Guifu Zhu, Ya Li

**Affiliations:** 1 Faculty of Information Engineering and Automation, Kunming University of Science and Technology, Kunming, China; 2 Information Construction Management Center, Kunming University of Science and Technology, Kunming, China; New York University Abu Dhabi, UNITED ARAB EMIRATES

## Abstract

Mainstream knowledge distillation methods primarily include self-distillation, offline distillation, online distillation, output-based distillation, and feature-based distillation. While each approach has its respective advantages, they are typically employed independently. Simply combining two distillation methods often leads to redundant information. If the information conveyed by both methods is highly similar, this can result in wasted computational resources and increased complexity. To provide a new perspective on distillation research, we aim to explore a compromise solution that aligns complex features without conflicting with output alignment. In this work, we propose to decouple the classifier’s output into two components: non-target classes learned by the student, and target classes obtained by both the teacher and the student. Finally, we introduce Decoupled Classifier Knowledge Distillation (DCKD), where on one hand, we fix the correct knowledge that the student has already acquired, which is crucial for merging the two methods; on the other hand, we encourage the student to further align its output with that of the teacher. Compared to using a single method, DCKD achieves superior results on both the CIFAR-100 and ImageNet datasets for image classification and object detection tasks, without reducing training efficiency. Moreover, it allows relational-based and feature-based distillation to operate more efficiently and flexibly. This work demonstrates the great potential of integrating distillation methods, and we hope it will inspire future research.

## Introduction

A powerful network is typically built upon a vast model foundation, but this also entails substantial computational costs. With the advancement of technology, such models are progressively transitioning towards being lightweight, with the goal of reducing costs. One potential direction for cost reduction is knowledge distillation (KD). KD refers to the process of aligning or approximating the logic or class predictions of a powerful, large-parameter teacher model on a smaller student model, given the same output [1, 2]. The aim is to achieve similar or better performance compared to the large teacher model. Thanks to the robust practical effectiveness of knowledge distillation, it has achieved tremendous success in multiple fields, including object detection [3], semantic segmentation [4], and transformer training [5].

Since the developments presented in [6], most current research has primarily focused on two directions: distilling knowledge from deep features and methods based on outputs. Feature knowledge distillation has proposed many methods in recent years 7,8, most of which are built on additional supervision of a pre-trained teacher model, especially the intermediate layers [6–12]. However, even though output-based distillation is less performant compared to feature knowledge distillation, it focuses on a deeper semantic level [13]. Both of these methods have their respective advantages. Feature distillation demonstrates significant performance benefits in various tasks, but due to its complexity, it incurs additional computational costs. On the other hand, output-based distillation requires comparatively fewer computational resources and storage costs, but its performance significantly lags behind that of feature distillation. The differences between the two methods are not just in terms of final performance and computational costs; the core distinction lies in the focus areas of the models they target. Generally, output-based distillation concentrates more on the real labels and the model’s outputs, whereas feature distillation emphasizes deep feature extraction. Each method has its own set of advantages that are difficult for the other to match. However, because they focus on different aspects, the knowledge learned by the student model also varies.

In the case of heterogeneous architectures between teacher and student models [14], the features of these models reside in different latent feature spaces, making it challenging to ensure effective alignment of the learned features. Consequently, directly matching these unrelated features is not only unproductive but may also hinder the student model’s performance. Furthermore, feature-based distillation methods focus more on local regions, and this localized attention may be insufficient for effectively transferring knowledge from the teacher model to the student model in knowledge distillation [15]. The knowledge embedded in the teacher model is often too complex for the student model to fully absorb and process [16]. Although teacher models are generally more complex and capable of capturing more knowledge, the main challenge lies in distilling this knowledge into a form that is accessible and beneficial for the student model [16].

We have observed that existing feature-based and output-based knowledge distillation techniques are mostly applied independently. Although both methods have their respective advantages in improving model performance and reducing model complexity, there is still no effective method that can simultaneously integrate the strengths of both, as they focus on different aspects of the model. As pointed out by Jianping Gou et al [17], how to model these different types of knowledge in a unified and complementary framework remains an urgent challenge. Specifically, knowledge from different layers may have varying impacts on the training of the student model [18]. For example, output-based knowledge mainly comes from the model’s final layer, while feature-based knowledge, guided by deeper layers, might face the risk of over-regularization. Meanwhile, recent studies have found that deep features are often linearized. Transforming features in certain directions can generate representations corresponding to the same category but with different samples. However, due to limited capacity, it is difficult for the student model to learn the distinctive features captured by the powerful teacher [19]. The lack of training data further exacerbates the performance gap between the student and teacher models.

Therefore, the key step in knowledge distillation lies in how to effectively transfer the rich knowledge of the teacher model to the student model. Based on this consideration, we propose a novel approach that decouples the classifier’s output from the distillation process by fixing the knowledge acquired through feature alignment and further mining the knowledge from the teacher model on both correct and incorrect categories to supplement the student’s learning. By simultaneously aligning the deep features and outputs of both the teacher and student models, we strive to minimize the performance gap between the two, and even surpass the teacher model in some experiments.

To achieve the above goals, we divide the structure into two core components: (1) retaining the target class knowledge already acquired by the student model, without fully covering all target classes; (2) utilizing both target and non-target class information to deeply align the teacher and student models. For better understanding, we refer to these two components as Stable Knowledge Distillation (SKD) and Polarized Knowledge Distillation (PKD), respectively.

**Fig 1:**
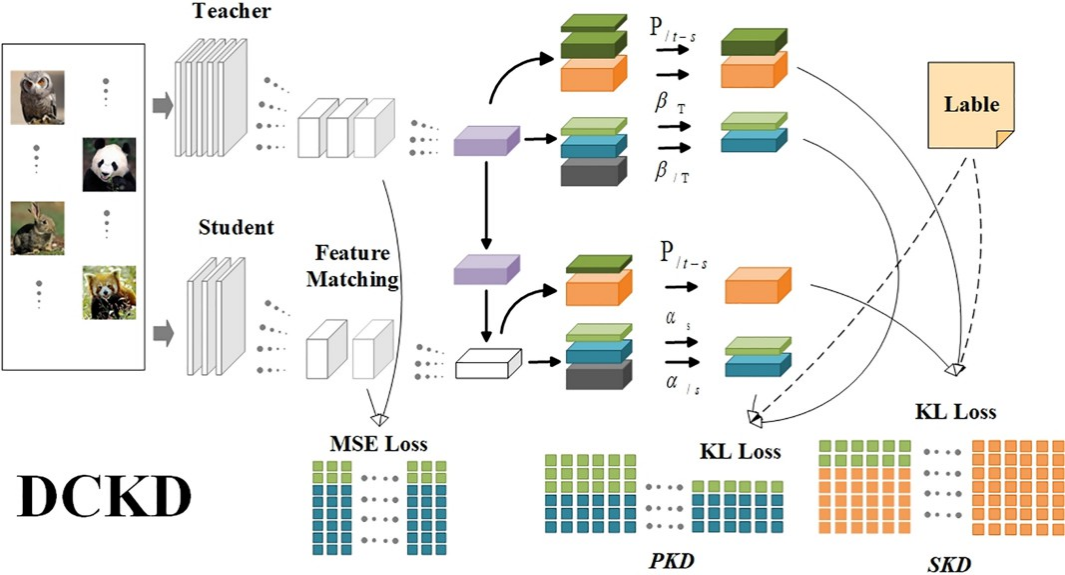
DCKD. Model architecture of DCKD.

Based on the aforementioned challenges, we propose an optimized output distillation method for feature-based knowledge distillation, called Decoupled Classifier Knowledge Distillation (DCKD), as illustrated in Fig 1. DCKD avoids the information redundancy caused by repetitive learning when using both feature-based and output-based distillation methods simultaneously. It also reinforces learned knowledge and excludes potential ambiguous or incorrect knowledge. The separate weighting of SKD and PKD helps determine the importance of each part.

In summary, our contributions are as follows:

(1)We decouple the classifier’s output into two parts, and split traditional KD into SKD and PKD. Through comparative analysis of these two components, we provide a suitable method to improve feature-based knowledge distillation by utilizing output-based distillation. (2)We reveal the limitations and even negative effects caused by redundant learning when combining output-based and feature-based distillation. PKD effectively avoids redundant learning of critical knowledge, while SKD further strengthens the knowledge based on PKD. (3)We propose a novel method, DCKD, that combines output-based distillation and feature-based distillation to complement each other and overcome these limitations. DCKD achieves state-of-the-art performance across various tasks.

## Related work

Knowledge distillation (KD) is a technique for transferring knowledge from a powerful teacher model, such as an ensemble of neural networks, to a smaller model [20–23]. This concept was first introduced by Hinton et al. [23], using the minimization of Kullback–Leibler (KL) divergence between logits to enhance the performance of the student model, primarily through the teacher model’s predictions or soft targets. The ultimate goal of KD is to counteract the decline in accuracy when compressing modelsl [20, 21], necessitating the extraction of as much information as possible from the teacher model, not only from the intermediate layers but also including the model’s representations [6, 23, 24]. Most current research focuses either on feature-based or output-based knowledge distillation, both achieving commendable results in relevant tasks. This includes leveraging intermediate feature maps and corresponding transformations [2, 6, 7, 24], cross logits [23–29], and mediator features [9, 10, 12, 13, 22–24, 30–37], with some recent studies shifting towards cross-layer correlations [8, 30]. Although output-based distillation performs lower than feature-based methods, its low computational and storage costs make it a method worth considering. While the most significant difference between these two methods lies in computational cost, our research finds that they fundamentally differ in the aspects of the model they focus on.

Since feature-based distillation methods focus more on learning the underlying characteristics of the model, while output-based methods typically emphasize the model’s response to true labels and final prediction performance, combining these approaches across multiple network layers can lead to information redundancy, causing an issue of repetitive learning. This repetitive learning not only fails to enhance knowledge transfer effectively but also adds computational complexity and potential error propagation to the model. Consequently, our research reveals that in feature-based knowledge distillation, further mining the teacher model’s knowledge is not simply a matter of stacking existing distillation frameworks or directly applying standard distillation strategies. Based on this insight, we propose a novel DCKD structure, whose core idea is to refine the knowledge transfer by using output-based distillation after aligning the student model’s features, thereby extracting more nuanced knowledge while effectively avoiding the negative impacts of repetitive learning. Experimental results show that the DCKD structure, despite its simplicity, achieves impressive outcomes in standard knowledge distillation settings, underscoring the effectiveness and innovation of our approach.

## Method

In this section, we will delve into the mechanisms of knowledge distillation. We have redefined the structure of the classifier’s output, dividing it into two parts: one related to the knowledge not yet learned, and the other related to the restructured knowledge. We explore the impact of each component within this framework and the limitations of traditional methods. Building on these studies, we propose a novel approach to output refinement, which has demonstrated excellent performance across various tasks.

### Distillation interference

The core objective of feature distillation is to minimize the mean squared error (MSE) between the intermediate features of the teacher model and the student model, formulated as follows:

$$\begin{array}{c} L_{\text{Feature}}=\|{\text{F}}^{\text{T}}-{\text{F}}^{\text{S}}{\|}_{2}^{2} \end{array}$$ (1)

In this process, the student model’s features F^S^ are adjusted to approximate the teacher model’s features F^T^. Once feature alignment is achieved, logits distillation is performed by further optimizing the output results through minimizing the KL divergence between the output probability distributions of the student and teacher:

$$\begin{array}{c} L_{\text{Logits}}=\text{KL}(\sigma ({\text{Z}}^{\text{T}}/\text{T})\|\sigma ({\text{Z}}^{\text{S}}/\text{T})) \end{array}$$ (2)

Here, Z^S^ and Z^T^ are the logits generated from the student’s features F^S^ and the teacher’s features F^T^ via the mapping function g, which typically consists of fully connected layers and nonlinear activation functions, converting the feature space into the logits space.

Although feature distillation and logits distillation act on different parts of the model (i.e., intermediate layers and output layers), there remains a dependency between the parameters and gradients of these two components. Adjustments to the intermediate layer parameters directly affect the output layer’s logits, and vice versa. Therefore, during logits distillation, in order to effectively minimize the KL divergence, the parameters of the student model’s output layer must be adjusted accordingly. Since the student’s logits Z^S^ are derived from the intermediate features F^S^ via the mapping function g, adjusting the output layer parameters may require the intermediate features F^S^ to be readjusted to fit the updated output targets.

During the optimization process of logits distillation, the student model’s parameter updates modify the input to the mapping function g, the intermediate feature representation F^S^. This adjustment may lead to feature shift, where the new feature representation ${\text{F}}_{\text{New}}^{\text{S}}$ differs from the original aligned F^S^ obtained through feature distillation:

$$\begin{array}{c} {\text{F}}_{\text{New}}^{\text{S}}\neq {\text{F}}^{\text{S}} \end{array}$$ (3)

This phenomenon is especially pronounced when the mapping function g has a complex structure and is highly sensitive to changes in intermediate features. The feature shift introduced by logits distillation may alter the features previously aligned during feature distillation, causing the value of ***L***_Feature_ to increase. This suggests that the adjustments made during logits distillation might negatively impact the knowledge acquired through feature distillation.

If the optimization of logits distillation causes F^S^ to be updated, it can be expressed as:

$$\begin{array}{c} \Delta {\text{F}}^{\text{S}}={\text{F}}_{\text{New}}^{\text{S}}-{\text{F}}^{\text{S}} \end{array}$$ (4)

If this change leads to an increase in ***L***_Feature_, it indicates that logits distillation has disrupted the original feature alignment, as represented by:

$$\begin{array}{c} \|{\text{F}}^{\text{T}}-({\text{F}}^{\text{S}}+{\text{F}}_{\text{New}}^{\text{S}}){\|}_{2}^{2}>\|{\text{F}}^{\text{T}}-{\text{F}}^{\text{S}}{\|}_{2}^{2} \end{array}$$ (5)

This implies that although logits distillation can effectively adjust the student model’s output to more closely match that of the teacher model, it may simultaneously disrupt the feature alignment in the intermediate layers, thereby impacting the knowledge previously obtained through feature distillation.

### Decomposition of classifier output

For a classification sample belonging to the t-th category, the classification probability can be represented as:

$$\begin{array}{c} \text{P}=[{\text{p}}_{1}+{\text{p}}_{2}+{\text{p}}_{1}+\ldots +{\text{p}}_{\text{t}}+\ldots +{\text{p}}_{\text{c}}]\in {\text{R}}^{1\times \text{C}} \end{array}$$ (6)

where p_*i*_ is the probability of the i-th category and c is the total number of class. Then, the softened probability of the i-th category in P after passing through the softmax function can be represented as:

$$\begin{array}{c} P_{i}^{s}=\frac{\text{exp}(\frac{\alpha_{i}^{s}}{T})}{\sum_{j=1}^{C}\text{exp}(\frac{\alpha_{i}^{s}}{T})} \end{array}$$ (7)

where T is a hyperparameter and $\alpha_{i}^{s}$ is the logit for the i-th category. *ε* = [p_t_, p_/t−s_] ∈ R^1×2^ represents the binary probability of the target category p_t_ and the rest of the non-target class not currently acquired by the student p_/t−s_. The calculation formulas are as follows:

$$\begin{array}{c} {\text{P}}_{\text{t}}^{\text{s}}=\frac{\text{exp}(\frac{\alpha_{\text{t}}^{\text{s}}}{\text{T}})}{\sum_{\text{j}=1}^{\text{C}}\text{exp}(\frac{\alpha_{j}^{s}}{\;\text{T}})} \end{array}$$ (8)

$$\begin{array}{c} {\text{P}}_{/\text{t}-\text{s}}^{\text{s}}=\frac{\text{exp}(\frac{\alpha_{/t-s}^{s}}{\;\text{T}})}{\sum_{\text{j}=1}^{\text{c}}\text{exp}(\frac{\alpha_{\text{j}}^{\text{T}}}{\text{T}})} \end{array}$$ (9)

The non-target probability for the student can be represented as:

$$\begin{array}{c} C_{P_{t}^{s}}=\frac{\text{exp}(\frac{\alpha_{i}^{s}}{T})}{\sum_{j=1,j\neq t-s}^{C}\text{exp}(\frac{\alpha_{j}^{s}}{T})} \end{array}$$ (10)

In this part, we attempt to split knowledge distillation (KD) into two components. One component is the target probability that the student has already acquired, and the other excludes the part of the target that has been learned $C_{P_{t}^{s}}$. Here, T and S, respectively, represent the teacher and the student. Traditional KD uses KL-Divergence as the loss function, which can be represented as:

$$\begin{array}{c} {\text{KD}}_{\text{KL}-\;\text{Divergence}\;}({\text{p}}^{\text{T}}\|{\text{p}}^{\text{S}})={\text{p}}_{\text{t}-\text{s}}^{\text{T}}\text{log}(\frac{{\text{p}}_{\text{t}-\text{s}}^{\text{T}}}{{\text{p}}_{\text{t}-\text{s}}^{\text{S}}})+\sum_{\text{j}=1,\text{j}\neq \text{t}-\text{s}}^{\text{C}}{\text{p}}_{\text{i}}^{\text{T}}\text{log}(\frac{{\text{p}}_{\text{j}}^{\text{T}}}{{\text{p}}_{\text{j}}^{\text{S}}}) \end{array}$$ (11)

The loss calculation for feature distillation uses the mean Squared error (MSE) loss function:

$$\begin{array}{c} {\text{KD}}_{\text{MSE}}=\frac{1}{\text{n}}\sum_{\text{i}=1}^{\text{n}}({\text{P}}_{\text{T}}^{\text{f}}-{\text{P}}_{\text{S}}^{\text{f}}{)}^{2} \end{array}$$ (12)

Due to the presence of multiple distillation methods, to avoid the impact of redundant learning on target probabilities, the loss function can be represented as:

$$\begin{array}{c} \text{KD}=\vartheta {\text{KD}}_{\text{MSE}}+\left(\vartheta /2\right){\text{KD}}_{\text{KL}} \end{array}$$ (13)

At the same time, only the probabilities of non-student targets are considered, which can be represented as:

$$\begin{array}{c} {\text{SKD}}_{{\text{C}}_{{\text{P}}_{\text{t}}^{S}}}=\sum_{j=1,j\neq t-s}^{C}p_{i}^{\text{T}}\;\text{log}(\frac{p_{j}^{T}}{p_{j}^{S}}) \end{array}$$ (14)

Additionally, as a complement to PKD, we restructured the outputs of the teacher and student models. Based on the target labels, we divide them into target and non-target classes *ε*_T_ and *ε*_S_, respectively, representing the probability distributions corresponding to the knowledge carried by the teacher and the student on the target class:

$$\begin{array}{c} \varepsilon_{\text{T}}=[\beta_{t},\beta_{/t}] \end{array}$$ (15)

$$\begin{array}{c} \varepsilon_{\text{S}}=[\alpha_{\text{t}},\alpha_{/t}] \end{array}$$ (16)

Therefore, considering only the currently acquired target and non-target classes, the outputs are reorganized into $P_{\beta_{t}\beta /t}$ and ${\text{P}}_{\alpha_{t}\alpha /t}$, according to Eq 7:

$$\begin{array}{c} {\text{OCRKD}}_{P_{t}^{s}}=P_{\beta_{t}\beta /t}^{T}\;\text{log}(\frac{P_{\beta_{\text{t}},\beta /t}^{\text{T}}}{{\text{P}}_{\alpha_{t},\alpha /t}}) \end{array}$$ (17)

In summary, DCKD can be represented as:

$$\begin{array}{c} \text{DCKD}=\sum_{j=1,\text{j}\neq t-\text{s}}^{\text{C}}{\text{p}}_{\text{i}}^{\text{T}}\;\text{log}\;(\frac{{\text{p}}_{\text{j}}^{\text{T}}}{{\text{p}}_{\text{j}}^{\text{S}}})+\left(\alpha /2\right){\text{P}}_{{\text{t}}_{\text{t}}\beta /t}^{\text{T}}\;\text{log}\;(\frac{{\text{P}}_{\beta \text{B}\beta }\text{T}/\text{t}}{{\text{P}}_{\alpha_{\text{t}}/\alpha /t}^{\text{T}}}) \end{array}$$ (18)

The above loss formula can then be expressed as:

$$\begin{array}{c} \text{KD}=\frac{\vartheta }{\text{n}}\sum_{\text{i}=1}^{\text{n}}({\text{P}}_{\text{T}}^{\text{f}}-{\text{P}}_{\text{S}}^{\text{f}}{)}^{2}+\left(\vartheta /2\right)(\sum_{j=1,j\neq t-s}^{\text{C}}{\text{p}}_{\text{i}}^{\text{T}}\;\text{log}\;(\frac{{\text{p}}_{\text{j}}^{\text{T}}}{{\text{p}}_{\text{j}}^{\text{S}}})+\left(\vartheta /4\right){\text{P}}_{\beta_{t},\beta /t}^{\text{T}}\;\text{log}\;(\frac{{\text{P}}_{\beta_{t}\beta /t}^{\text{T}}}{{\text{P}}_{\alpha_{t},\alpha /t}^{S}})) \end{array}$$ (19)

The above insights inspire us to explore the individual effects of SKD (Soft Knowledge Distillation) and PKD (Probabilistic Knowledge Distillation), which will aid in further mining knowledge from the teacher model.

### Effects of SKD and PKD

To better demonstrate the effectiveness of DCKD, we studied the impact of SKD and PKD on CIFAR-100, using ResNet [38], WideResNet(WRN) [12], and ShuffleNet [39] as training models. We thoroughly considered the effects of both similar and dissimilar architectures. The experimental results are shown in Table 1. For each teacher–student pair in the table, we display the baseline performance of the student (standard training based on logits), experiments using SKD alone, experiments using PKD alone, and experiments using both simultaneously. The training weights are set to 0.5, 1, and 1, corresponding to PKD, SKD, and Feature, In this Feature method, MSE loss is used to align the features, and the final layer is reused to further align feature knowledge, following the setup referenced from SimKD [8], respectively, with the implementation in Section 4 also following this setup, and set to 0.5.

**Table 1:** Accuracy (%) on the CIFAR-100 validation set. Δ Represents performance improvement relative to the baseline. The bold sections represent the results of using our method.

Student	PKD	SKD	Top-1	Δ
ResNet32x4 as the Teacher 79.41				
ResNet8x4 73.09			77.92	-
		√	77.93	0.01
	√		77.85	-0.07
	√	√	78.47	0.55
ShuffleNet-V1 71.36			77.12	-
		√	77.14	0.02
	√		76.56	-0.56
	√	√	77.79	0.67
WRN-40-2 as the Teacher 76.31				
ResNet8x4 73.09			76.55	-
		√	76.57	+0.02
	√		76.41	-0.14
	√	√	76.9	0.35
ShuffleNet-V1 71.36			77.02	-
		√	77.03	0.01
	√		76.8	-0.22
	√	√	77.38	0.36

Intuitively, SKD focuses on knowledge that has not been correctly processed, which includes both the target and non-target classes. PKD, on the other hand, targets redundant learning, preserving knowledge that has already been correctly processed to avoid relearning.

As shown in Table 1, under different teacher models, SKD and PKD exhibit varying effects, with some showing improvements and others showing declines. However, it is only when both are applied simultaneously that the overall results show a consistent improvement, surpassing the effects of each used individually. For example, when WRN-40-2 is the teacher and ResNet84 is the student, PKD alone shows a modest gain of 0.27, but the maximum gain of 0.35 is only achieved when both are working together. Moreover, compared to traditional KD (as shown in Table 2), there is a clear advantage. The results indicate that SKD and PKD function as inseparable components, complementing each other to maximize the extraction of knowledge that may be lacking during the knowledge transfer between teacher and student models. To delve deeper into this finding, we will proceed with a detailed analysis.

**Table 2:** Accuracy (%) on the CIFAR-100 validation set. Δ Represents performance improvement relative to the baseline. The bold sections represent the results of using our method.

Student	Feature	Logits	PKD
ResNet32x4 as the Teacher 79.41			
ResNet8x4 73.09	77.92	76.01 (-1.91)	77.85 (-0.07)
ShuffleNet-V1 71.36	77.12	74.78 (-2.64)	76.56 (-0.56)
WRN-40-2 as the Teacher 76.31			
ResNet8x4 73.09	76.55	74.46 (-2.09)	76.41 (-0.14)
ShuffleNet-V1 71.36	77.02	76.55 (-0.47)	76.8 (-0.22)

SKD serves to retain the accurate knowledge obtained from feature alignment. According to Eq 17, DCKD fixes this knowledge by restructuring the output. To validate SKD’s effectiveness in suppressing repetitive learning. These experiments Table 1 aim to demonstrate that traditional, undifferentiated KD is incompatible with the current feature-based knowledge distillation approach.

As shown in Table 2, combining either traditional KD methods or our PKD approach directly with current feature-based knowledge distillation leads to a significant performance drop. This decline even exceeds previous improvements; for example, when using ResNet324 as the teacher model and ResNet84 as the student model, applying traditional KD for output distillation results in a substantial performance decrease of 1.91. According to the formula, SKD prunes portions of knowledge to avoid redundant learning of already mastered information. In contrast, traditional KD and PKD lack adaptive adjustments, resulting in redundant knowledge learning. Output-based distillation and feature-based distillation focus on different aspects of the model: the former primarily targets the output layer, while the latter emphasizes the underlying structure.

The DCKD method enhances the learning capacity of the student model by adjusting the output structure to incorporate knowledge that has not been fully processed into the distillation process. According to the results in Table 2, SKD performs excellently in most scenarios, but when combined with PKD, the overall model performance is further improved. This improvement is evident across multiple teacher-student model combinations, particularly when WRN-40-2 is used as the teacher model and ResNet8x4 or ShuffleNet-V1 as the student model, where performance varies in degree.

Using SKD or PKD alone has limited effect and does not significantly improve model performance, but when combined, performance is noticeably enhanced, demonstrating their synergistic effect in knowledge extraction and transfer. As shown in Eq 8, SKD decouples the output to provide a clearer knowledge structure for the student model’s learning. It also introduces the same pruning operation in both the student and teacher models to prevent redundant learning content during knowledge transfer. This pruning process is specifically optimized for the student model to ensure the efficiency and uniqueness of acquired knowledge, thereby further improving the overall effectiveness of knowledge distillation.

### Decoupled Classifier Knowledge Distillation

Up to this point, we have redefined the output of the classifier as a weighted sum of two independent parts and further validated the effectiveness of SKD, revealing the superiority and importance of PKD. Whether on the CIFAR-100 dataset or the more challenging ImageNet dataset, DCKD has achieved significant improvements. Furthermore, our experiments have shown that the impact of SKD and PKD is non-negligible. Their respective roles complement each other, and only by combining them can we maximize the coverage of the knowledge gaps that exist in the knowledge transfer between different teacher–student pairs.

ResNet18 contains a significant number of convolutional layers. For student models such as ResNet116, the internal structure is even more extensive, reaching a remarkable 116 layers. In more advanced teacher models like ResNet110x2, the number of convolutional layers amounts to as many as 220. Each convolutional operation results in a transformation of the parameter dimension.

In convolution operations, if the stride is greater than 1 or padding is not used, the spatial resolution of the output feature map decreases, leading to the loss of some spatial information. Since the convolutional kernel covers only a small local region of the image, each convolutional operation captures only local, not global, information. Furthermore, activation functions (like ReLU) can cause information loss as they turn all negative inputs to zero. Pooling layers (such as max pooling) also lead to information loss by reducing the dimensions of the feature map through downsampling.

We attempt to restructure the student model to retain as much of its learned target knowledge as possible. Based on relevant formulas, we reconstructed the student model’s output to focus more on the knowledge transferred from the teacher and the true labels. The teacher and student share the same network structure, with the primary difference being the number of layers in each. Due to the significant loss of features across multiple processing stages, the differences between teachers and students with varying sizes or network structures become more pronounced.

**Fig 2:**
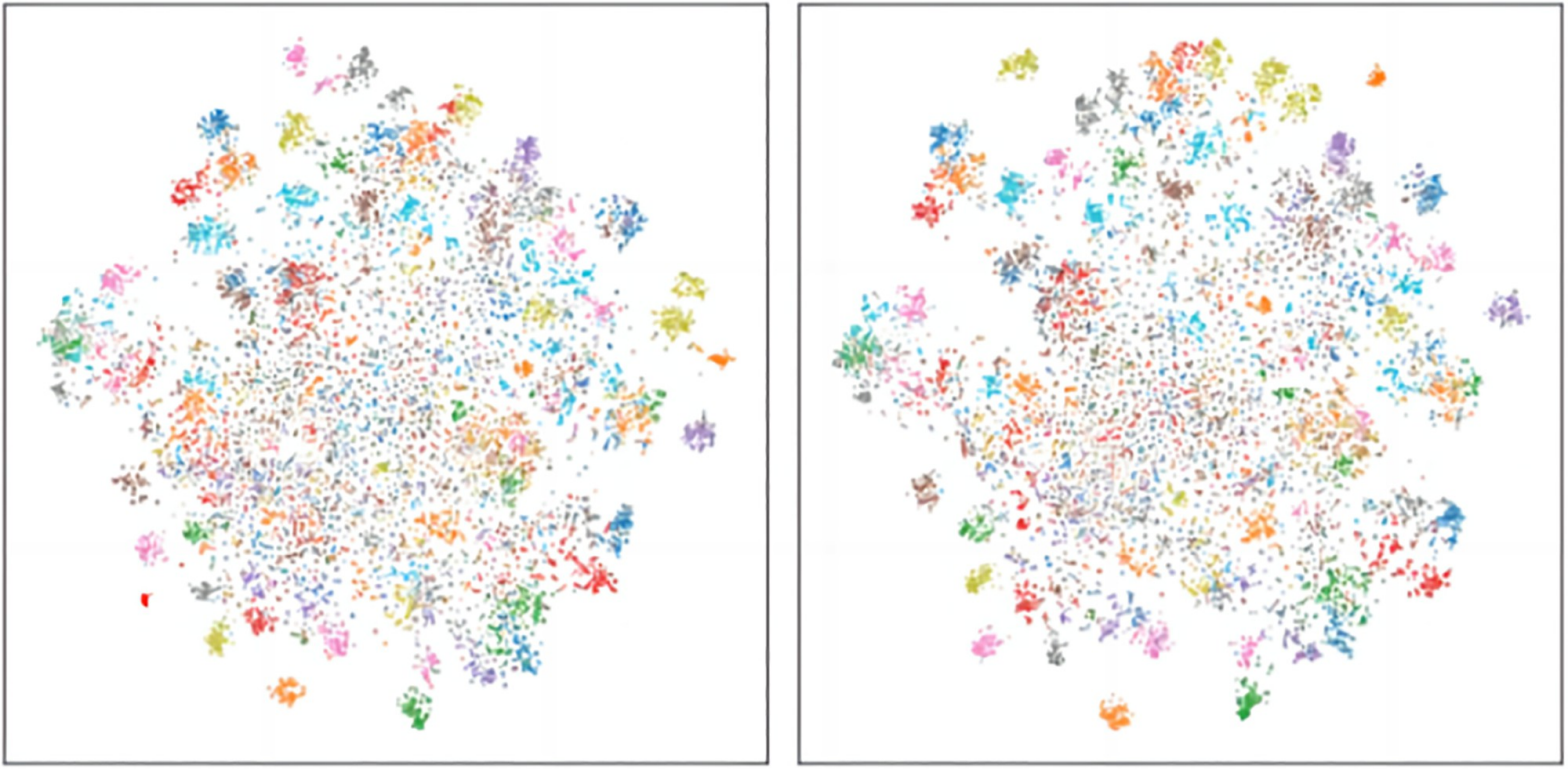
The t-SNE visualization of features. The improved scatter plot using our method (left) and the original scatter plot of Feature (right).

The Fig 2, using the T-SNE visualization technique, displays the effects of different knowledge transfer methods. The left image shows the clustering result after using the DCKD method. The distribution is more compact, forming distinct clusters, indicating a higher similarity between data points. This tight clustering suggests that the features extracted using the DCKD method better reflect the intrinsic connections between data points.

**Fig 3:**
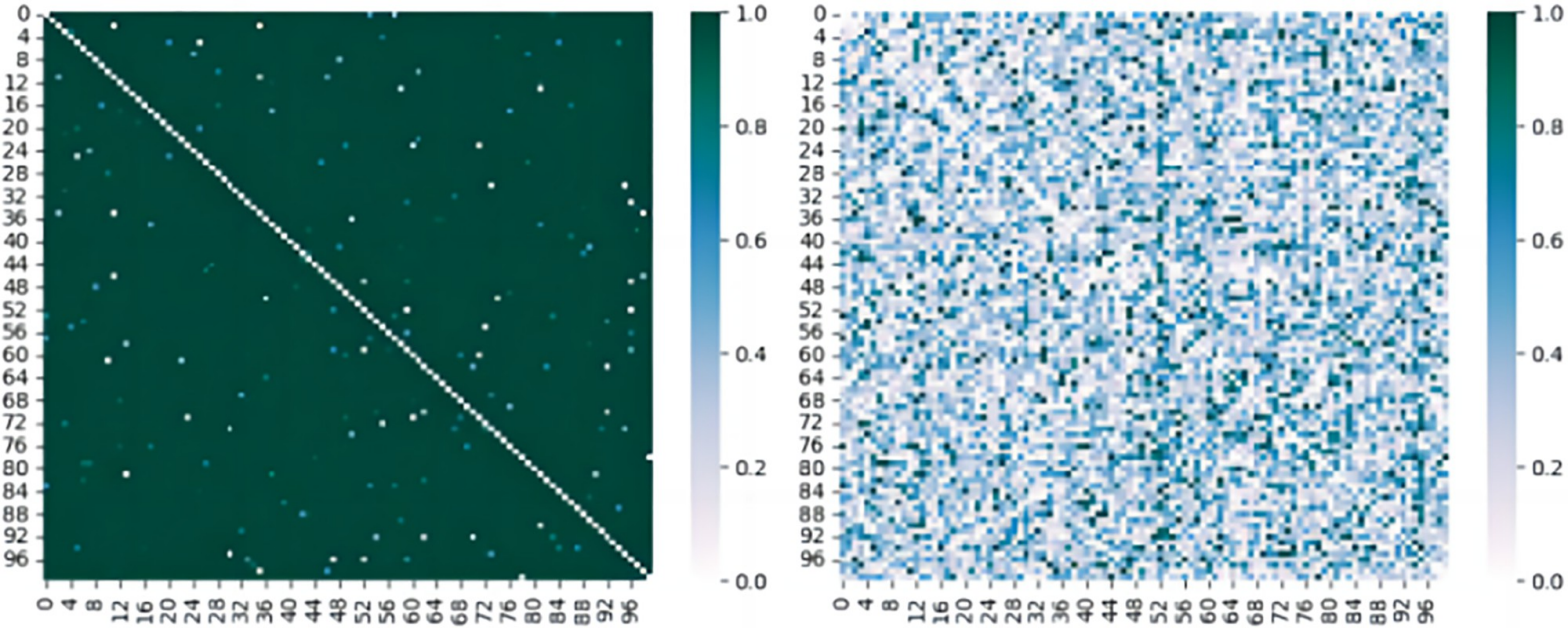
The improved similarity matrix using PKD (left) and the original scatter plot of DCKD (right).

The right image shows a relatively looser distribution. While clustering is still observable, the boundaries of these clusters are not as clear-cut as in the left image. This indicates that the Feature method retained more variability in the feature space but failed to learn sufficient features. It can also be observed that, with the filtering of DCKD, the acquired knowledge is effectively consolidated, with certain positions remaining unchanged. Comparative analysis shows that the DCKD method has a distinct advantage in knowledge transfer; it is able to further convey the teacher model’s useful knowledge to the student model while preserving knowledge unique to the student. This results in the student model becoming more focused on certain feature positions, while more dispersed in others. To demonstrate that the student retains its unique knowledge, we have plotted the similarity matrices of the two methods, as shown below Fig 3:

These two Fig 3 are visualizations of similarity matrices, comparing the similarity between data points. In the matrix on the left, we see a dark-colored background with bright spots along the diagonal, indicating perfect similarity of each element with itself. Few other bright spots appear in the matrix, suggesting that most elements have low similarity to each other, with only a few element pairs showing higher similarity. The matrix on the right illustrates a more complex scenario. Its diagonal also shows high self-similarity for each element, but the color variations within the matrix are more prominent, indicating a broader distribution of similarity across data points. In this figure, the differentiation of similarities is more evident, displaying a range of high and low similarities between data points, which may suggest a more complex data structure with richer internal characteristics. This interpretation is further supported in the subsequent experimental section.

From these two matrices, we can conclude that in the first matrix, most data points are relatively independent, with only a few notable similarity relationships. In contrast, in the second matrix, the similarity of data points is more dispersed, revealing a relatively chaotic internal structure. Therefore, we conclude that using PKD alone may disrupt the learned feature knowledge, even causing the student model to diverge significantly from the teacher model. Additionally, we designed a simple experiment to verify the rationality of the weight settings.

From the above Table 3, it can be observed that setting weights to 1 and 0.25 leads to varying degrees of performance decline. We believe that excessively high weights can alter the learning focus, while overly low weights fail to fully demonstrate the effectiveness of the method.

**Table 3:** Results on the CIFAR-100 dataset. We tested the results with different values of: 1, 0.5, and 0.25.

Model	ResNet32x4 79.41	ResNet32x4 79.41	ResNet32x4 79.41	WRN-40-2 76.31
	ResNet8x4 73.09	ShuffleNet-V1 71.36	ShuffleNet-V2 72.6	WRN-40-1 71.92
*θ* = 1	78.23	77.3	77.43	75.2
*θ* = 0.5	78.47	77.79	78.1	75.89
*θ* = 0.25	78.39	77.59	77.65	75.5

## Experiments

Experiments were conducted on two representative tasks: object detection and image classification, which included CIFAR-100 [40] and ImageNet [41]. CIFAR-100 is a well-known image classification dataset comprising 32 × 32 images across 100 classes. ImageNet, on the other hand, is a large-scale classification dataset with 1000 classes, including 1.28 million images in the training set and 50,000 images in the test set.

### Main results

This section will elaborate in detail the improvements achieved using DCKD and validate the performance of our proposed method in image classification and object detection tasks through benchmark testing. To test our DCKD, we conducted experiments on CIFAR-100 and reported the accuracy of the experimental results. For comparison, we used various methods, including FitNet [6], AT [42], SP [10], VID [7], CRD [9], SRRL [11], and SemCKD [8].

We followed the training procedures of previous research [8, 9, 11] and reported the performance of all competitors on randomly associated teacher–student combinations. Specifically, we used the SGD optimizer, with the Nesterov momentum set to 0.9 for all datasets. For CIFAR-100, the total training epochs were set to 240, with the learning rate divided by 10 at epochs 150, 180, and 210. The initial learning rate was set to 0.01 for MobileNet/ShuffleNet architectures and 0.05 for other architectures. The size of each mini-batch was set to 64, and the weight decay was set to 5 × 104. For ImageNet, the initial learning rate was set to 0.1, which was then divided by 10 at epochs 30, 60, and 90 of the 120 training epochs. The mini-batch size was set to 256, and the weight decay was set to 1 × 104. All results are presented as the mean (standard deviation) of four trials, except for ImageNet results, which are based on a single trial. In KD loss, the temperature T was consistently set to 4.

### Extensions

Next, we will set up 15 different combinations of teacher and student models to compare their performance on the CIFAR-100 dataset. To demonstrate the versatility and cross-model capabilities of our method, we referred to ten different distillation approaches, including our own. These combinations encompass both similar and completely different network architectures for the teacher and student models.

Table 4 demonstrates that when the teacher and student models adopt the same network structure, our method shows a significant advantage over all competitors, achieving a leading result. Particularly notable is when the teacher model is ResNet32x4 and the student model is ResNet8x4, where the performance of the model breaks through 78%, reaching 78.47%, surpassing the nearest competitor by 0.55. In the experiments with the derived model WRN, our approach similarly achieves a performance advantage, exceeding the nearest competitor by 0.33. Through comparative experiments, we further demonstrate that the DCKD model, while maintaining generalizability, achieves higher accuracy. Our model exhibits superior performance in various scenarios, thanks to our balanced approach in the training process, which considers both the teacher model and maintaining generalizability.

**Table 4:** Results on the CIFAR-100 dataset. We tested the results with different values of: 1, 0.5, and 0.25.

Teacher	ResNet32x4 79.41	WRN-40-2 76.31	ResNet110x2 78.18	ResNet110x2 78.18
Student	ResNet8x4 73.09	WRN-40-1 71.92	ResNet110 74.37	ResNet116 74.46
KD [31]	73.08	73.54	76.25	76.14
FitNet [6]	74.48	74.23	75.84	76.07
AT [12]	75.24	74.82	76.89	76.92
SP [10]	74.47	74.22	76.51	76.68
VID [7]	75.04	74.52	76.6	76.38
CRD [9]	75.54	74.92	76.63	76.93
SRRL [11]	75.46	74.73	76.72	76.5
SemCKD [8]	75.86	74.75	77.02	77.1
SimKD [43]	77.96	75.56	77.61	77.95
DCKD	78.47 (+0.51)	75.89 (+0.33)	77.73 (+0.12)	77.84 (-0.11)

Tables 5 and 6 demonstrates that when the teacher and student models adopt the different network structure, our method achieves excellent performance regardless of the network construction of the teacher and student models. Particularly notable are the scenarios where the teacher model is WRN-40-2 and the student model is ResNet8x4, where the teacher model is ResNet32x4 and the student model is WRN-40-2, and where the teacher model is ResNet110x2 and the student model is ShuffleNet-V2x1.5. In these instances, the student models not only surpassed their respective teacher models but also approached an accuracy close to 80. The performance of the teacher and student models in these cases are 76.31/76.9, 79.42/79.64, and 78.18/79.02, respectively.

**Table 5:** Results on the CIFAR-100 dataset. The teacher model and the student model come from the different structure.

Teacher	ResNet32x4 79.41	ResNet32x4 79.41	ResNet32x4 79.41	ResNet32x4 79.41	ResNet110x2 78.18
Student	ShuffleNet-V1 71.36	ShuffleNet-V2 72.6	VGG8 70.46	MobileNet-V2x2 69.06	ShuffleNet-V2x1.5 74.15
KD [31]	74.07	74.45	72.73	72.43	76.82
FitNet [6]	74.97	75.48	72.88	65.98	66.25
AT [12]	75.69	76.25	72.46	66.25	78.31
SP [10]	75.59	76.18	73.08	61.23	77.76
VID [7]	75.47	75.89	73.02	65.41	77.92
CRD [9]	75.45	76.2	73.06	66.62	78.41
SRRL [31]	75.13	76.27	73.5	65.48	78.54
SemCKD [8]	76.97	77.59	74.96	68.72	78.87
SimKD [43]	77.18	77.67	75.29	75.42	78.81
DCKD	77.79 (+0.61)	78.1 (+0.43)	75.61 (+0.32)	73.32 (-2.1)	79.02 (+0.21)

**Table 6:** Results on the CIFAR-100 dataset. The teacher model and the student model come from the different structure.

Teacher	WRN-40-2 76.31	WRN-40-2 76.31	ResNet32x4 79.41	ResNet32x4 79.41	ResNet110x2 78.18
Student	MobileNet-V2 65.43	ResNet8x4 73.09	WRN-16-2 73.51	WRN-40-2 76.31	ShuffleNet-V2 72.6
KD [31]	69.07	75.28	74.9	77.7	76.05
FitNet [6]	68.63	75.12	74.51	77.8	76.03
AT [12]	68.62	75.72	75.29	78.94	76.54
SP [10]	68.63	74.22	75.47	78.19	77.67
VID [7]	69.03	75.38	75.29	77.99	76.83
CRD [9]	70.49	75.88	75.97	78.49	76.92
SRRL [11]	70.2	75.66	75.53	78.3	77.34
SemCKD [8]	69.92	75.16	74.96	78.06	77.44
SimKD [43]	70.34	76.55	77.07	79.13	77.72
DCKD	70.59(+0.25)	76.9(+0.1.39)	77.32(+0.25)	79.64(+0.51)	77.83(+0.11)

Table 7 shows the Top-1 accuracy and Top-5 accuracy of different methods, and our method is still in the leading position. Our method demonstrates strong adaptability and effectiveness in handling knowledge transfer between teacher and student models of different network structures. This not only validates the correctness of our method but also provides robust support for its scalability and flexibility in practical applications. At the same time, we verified the results under the ImageNet dataset (as shown in Table 8), which showed remarkable results even in the face of large data sets.

**Table 7:** Results on the CIFAR-100 dataset.displays the Top-1 and Top-5 accuracies for different methods on the CIFAR-100 dataset. Our method achieved excellent results across various competitors.

Distillation Method	Top-1	Top-5
KD [31]	73.08	89.88
FitNet [6]	74.48	93.34
AT [12]	75.24	93.65
SP [10]	74.47	93.47
VID [7]	75.04	93.68
CRD [9]	75.54	93.8
SRRL [11]	75.46	93.83
SemCKD [8]	75.86	93.89
SimKD [43]	77.92	94.1
DCKD+	78.47 (+0.55)	94.46 (+0.35)

**Table 8:** Results on the ImageNet.shows the performance on the ImageNet dataset, where we referenced three different methods. DCKD achieved advanced results across students of varying sizes. Additionally, to better illustrate these outcomes, we supplemented with more experiments to demonstrate the specificity of DCKD in knowledge processing.

Teacher	ResNet50	ResNet50
Student	ResNet34	ResNet18
SimKD [8]	74.64	72.52
NormKD [44]	73.94	72.33
Ours	75.07 (+0.43)	72.68 (+0.14)

**Fig 4:**
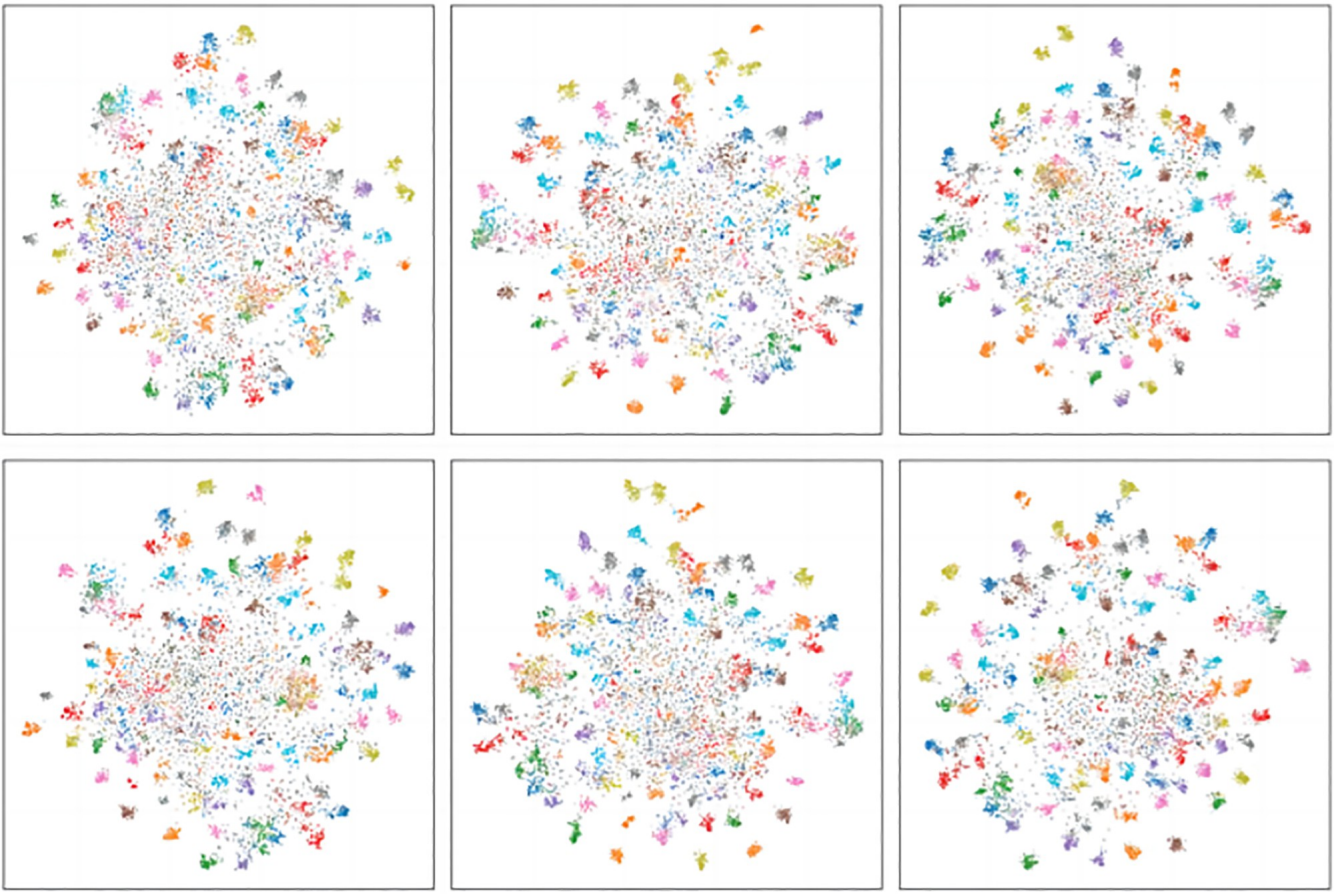
The image above shows the T-SNE plot of the features learned by students, with the top row representing DCKD and the bottom row representing SimKD. From left to right, the combinations of teacher and student models are as follows: ResNet32x4 and ShuffleNet-v1l; ResNet38x2 and ResNet8x4; and ResNet38x2 and ShuffleNet-v1.

Fig 4 displays the visualization results of features learned by different methods. In the top image (DCKD), the data points exhibit a clear clustering structure, distinctly distributed across different regions, indicating a strong separability of various data points in the feature space. This clustering effect suggests that our method effectively preserves and reveals the structural information of the original data during the distillation process for high-dimensional data. Simultaneously, the color distribution forms clear boundaries between clusters, highlighting significant differences between features of different classes. Through this effective differentiation, our method enables the student model to align more closely with the teacher model in feature representation, thereby providing greater accuracy for subsequent classification tasks.

In contrast, the image on the right shows the clustering effect of another method. While a clustering pattern is also present, the boundaries between clusters are blurred. Compared to the left image, this blurred boundary may indicate that this method has lost some detailed information during knowledge distillation, leading to reduced differentiation between categories. This effect may stem from the method’s insufficient utilization of the structured knowledge transferred from the teacher model, resulting in a lack of inter-class feature distinction in the student model. Thus, this comparison further demonstrates the effectiveness of the DCKD method in preserving the original feature structure and enhancing classification accuracy.

### Discussion and conclusions

This paper reconstructs the classifier output through an in-depth analysis of the classifier and proposes a novel knowledge distillation loss calculation method, providing a clearer explanation for output-based knowledge distillation. This innovative perspective offers new theoretical support for understanding the internal mechanisms of knowledge distillation while expanding the potential for developing distillation techniques. For the first time, this approach combines feature-based knowledge distillation with a new distillation loss, not only improving model performance but also providing new research directions for tackling more complex tasks. Through this innovative integration, the student model can better leverage the deep-level knowledge provided by the teacher model during the learning process.

However, despite the promising results demonstrated in experiments, there are some limitations that warrant further investigation. First, effectively extracting feature knowledge from the teacher model remains challenging in mainstream tasks like computer vision. While current feature distillation methods can partially utilize the deep information from the teacher model, they struggle to comprehensively capture all valuable knowledge. Additionally, tasks such as image detection and object recognition are computationally intensive, and the distillation process often increases the computational demand during model training. Therefore, a key issue to address in the future is how to maintain model performance while reducing computational costs.

Another noteworthy limitation is the high computational cost of existing distillation methods when applied to large-scale datasets, especially when handling high-dimensional and multimodal data, where efficiency challenges become more apparent. While distillation techniques can reduce model size and accelerate inference, further improvements in distillation efficiency and optimization of the student model’s learning process are needed for larger-scale, more complex scenarios. Additionally, although the proposed distillation loss offers interpretability for classifier outputs, its sensitivity to specific tasks may vary, implying the need for customized loss designs across different tasks.

To address these limitations, future research will further explore ways to extract knowledge from teacher models more efficiently and enable the student model to benefit from it. We plan to introduce advanced feature extraction and fusion techniques, such as multi-scale feature processing and cross-layer feature capture, to acquire richer knowledge. We will also explore adaptive adjustments to the distillation loss function for different tasks to enhance generalizability and flexibility. Furthermore, optimizing the computational efficiency of the distillation process is a priority, including techniques such as model pruning, quantization, and selective knowledge distillation to reduce unnecessary computational overhead.

Looking ahead, we aim to design lightweight student model architectures to achieve high accuracy with significantly reduced computational costs. For multimodal data processing, we will further investigate how to effectively integrate information from different modalities to enhance the overall performance of the student model. By continuing to explore and innovate on these key issues, we hope to achieve further breakthroughs in the field of knowledge distillation, promoting its application and adoption across a wider range of practical scenarios.
